# Medical Students’ Perspectives Regarding the Use of a Slit-Lamp Smartphone Adapter for Clinical Slit-Lamp Photography

**DOI:** 10.7759/cureus.57986

**Published:** 2024-04-10

**Authors:** Mohammed M Emam, Meshari A Alharbi, Abdulaziz Alammar, Mohammad I Aldekhail, Abdulrahman Alammar, Omar Solyman, Ahmed M Alaraj

**Affiliations:** 1 Department of Radiologic Technology, College of Applied Medical Sciences, Qassim University, Al-Qassim, SAU; 2 Department of Ophthalmology, Qassim University, Al-Qassim, SAU; 3 Department of Ophthalmology, King Fahad Specialist Hospital, Buraydah, SAU; 4 Department of Ophthalmology, Qassim University Medical City, Al-Qassim, SAU; 5 Department of Ophthalmology, College of Medicine, Qassim University, Al-Qassim, SAU

**Keywords:** prospective study, outcome assessment, electronic medical record (emr), digiscoping, smartphone camera, slit-lamp

## Abstract

Background

This study aimed to investigate medical students’ perspectives regarding the ease and utility of smartphone slit-lamp photography.

Methodology

In this prospective experimental study, fourth and fifth-year medical students who were in or had finished ophthalmology rotation were included to attempt slit-lamp smartphone anterior segment photography on adult patients after a brief hands-on instruction course. Each medical student attempted to record five supervised slit-lamp videos of the anterior segment of five patients using the described adapter and their own smartphone. The time taken until photography was calculated for each attempt. After the fifth attempt, each medical student rated the ease of the use of this method of slit-lamp photography as well as their perspective regarding its utility as a potential means of medical education and telemedical consultations on a five-point Likert scale.

Results

A total of 33 medical students participated, with each successfully recording five slit-lamp examinations using their smartphones. The time used for the application of the adapter until the image capture ranged from 6 to 278 seconds (average = 39.51 ± 34.7 seconds) and markedly improved by the fifth attempt (30.5 ± 25.7 seconds) compared to the first attempt (67.3 ± 49.3 seconds). Learning this skill was perceived to be relatively easy (2.2 ± 1), with high potential in clinical education (4.6 ± 0.75) and teleconsultations (4.7 ± 0.65).

Conclusions

Smartphone slit-lamp photography is a relatively easy process. It can be quickly acquired by medical students and has the potential to enhance their medical education and telemedical consultation capabilities.

## Introduction

Slit-lamp photography plays an essential role in clinical documentation, medical education, telemedical consultation, and clinical research. Slit-lamp photography is classically performed by a permanently mounted camera, which often requires a computer system for data storage and network connectivity [1]. In addition, such mounted slit-lamp cameras require a significant amount of clinic space, are stationery that limits their use to a single examination lane, and require operation by trained technicians or physicians [2].

The ubiquity and ever-ready presence in addition to the high level of connectivity, portability, and multi‑functionality of smartphones make them an important and widely used tool for ophthalmic clinical photography [3-6]. Smartphone camera use for slit-lamp photography has been proven effective and reliable for the documentation of different ocular conditions, including assessment of glaucomatous optic neuropathy [7], grading of diabetic retinopathy [8], evaluation of senile cataracts [9], and postoperative cataract surgery imaging [4].

For clinical photography through the slit lamp, the digital camera needs to be perfectly aligned with the optical center of the slit-lamp ocular [10]. Different adapters have been described for this alignment, some of which are custom-designed, and others are commercially available smartphone adapters. Available smartphone adapters are often slit lamp and smartphone specific, which means they are compatible with certain kinds of slit lamps and specific smartphone versions [5,11]. In addition, the application and disassembly to and from the slit lamp are usually tedious and time-consuming [1]. Here, we describe and validate the use of a novel design of a smartphone slit-lamp adapter from medical student perspectives in terms of its time efficiency, ease of use, and perceived value in medical education and potential medical consultations.

## Materials and methods

This prospective, experimental study was approved by the institutional review board of Qassim University, Al-Qassim, Saudi Arabia (approval number: 23-50-02) and was performed following all local laws and compliance with the principles of the Declaration of Helsinki. Fourth and fifth-year medical students who had finished ophthalmology rotation or were currently in ophthalmology rotation and accepted to participate in the study were included to attempt slit-lamp smartphone photography. Medical students who had not yet had their ophthalmology rotations were excluded. Adult patients in the ophthalmology outpatient clinic with anterior segment abnormality amenable to photography were included to undergo slit-lamp photography. Patients who could not be seated comfortably on the slit lamp and those who were photophobic to the slit-lamp light were excluded. Written informed consent was obtained from all participating patients and oral consent was obtained from all participating medical students.

**Figure 1:**
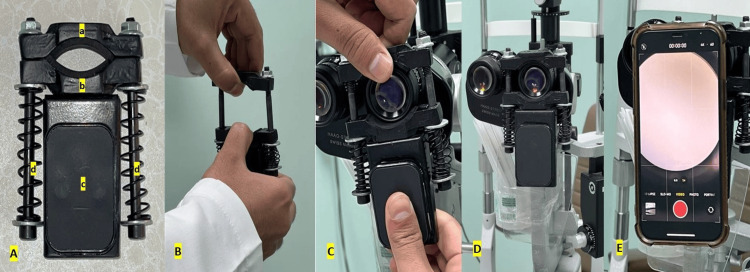
An external image demonstrating the application process of the smartphone slit lamp adapter for digital clinical photography. An external image showing the composition of the smartphone slit-lamp adapter and the steps of its application to the slit lamp for photography. (A) The adapter. a and b: the upper and lower ring plates which encompass the slit lamp adapter, c: the magnet, d: metal springs with counteraction retaining the adapter in place after application. (B) Spreading the ring plates apart. (C) Accommodating the ocular of the slit lamp between the ring plates. (D) The adapter is self-retained on the ocular of the slit lamp after release of the pull on the ring plates. (E) The smartphone camera is turned on and the smartphone is applied to the magnetic part of the adapter following the light circle emanating from the ocular of the slit lamp. (F) The smartphone snaps to the adapter and remains steady and ready for digital photography.

Participating medical students underwent an additional hands-on instruction course on basic techniques of using the slit lamp and on the application of the adapter. Each participating medical student was then required to record five supervised slit-lamp videos of the anterior segment of five different patients using the described adapter and their own smartphone. The recording process started after the formal examination of the patient was performed. The patient was asked to stay seated on the slit lamp with the slit lamp focused on the anterior segment under diffuse illumination and the magnification set at 10×. The slit lamp was kept steady in place by tightening the stabilization screw. The application time was counted starting from the moment the medical student started the application of the adapter on the slit lamp by snapping the phone over the magnet of the adapter and adjusting the view until a focused view of the diffusely illuminated anterior segment came into view (Figure 1, Video 1). After medical students had completed their five attempts, they were asked to rate the ease of the use of the adapter for slit-lamp photography on a scale of 1 to 5, with 1 being very easy and 5 being extremely difficult. Participants were also asked to rate their perspectives on the utility of the method using this adapter for smartphone slit-lamp photography as a potential means of medical education and telemedical consultations on a scale of 1 to 5, with 1 being useless and 5 being extremely useful.

**Video 1:** An external video recording demonstrating the application process of the smartphone and adapter to the slit lamp for slit-lamp photography.

Data was fed to the computer and analyzed using SPSS version 20.0. (IBM Corp., Armonk, NY, USA). Categorical data were represented as numbers and percentages. Continuous data were tested for normality by the Shapiro-Wilk test. Quantitative data were expressed as range (minimum and maximum), mean, standard deviation, and median. Friedman test was used to detect differences in durations across multiple test attempts, and then the post hoc test was used to determine if these differences were significant. Statistical significance was considered at p-values ≤0.05.

## Results

**Figure 2:**
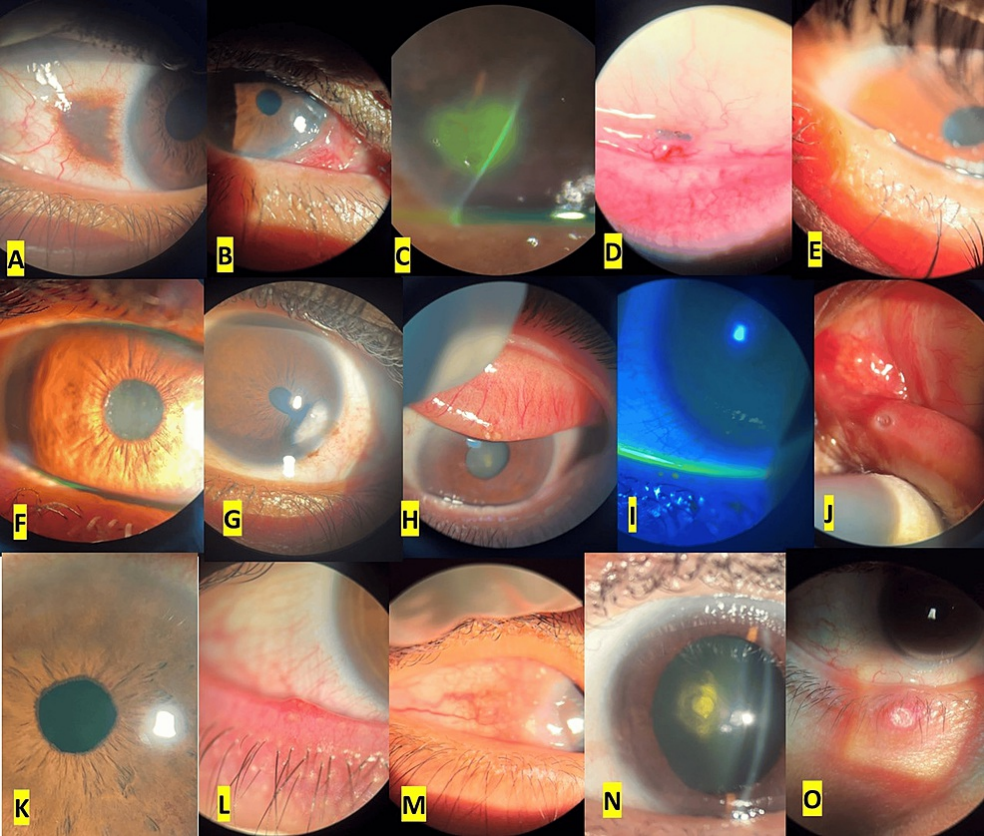
Samples of digital clinical smartphone slit-lamp photographs. (A) Conjunctival nevus. (B) Limbal conjunctival epibulbar mass. (C) Fluorescein staining of a corneal ulcer. (D) Inferior fornical conjunctival foreign body. (E) Meibomian gland dysfunction with a retention cyst. (F) Pseudoexfoliation syndrome. (G) Corneal scarring with peaking of the pupil toward the scar. (H) Superior tarsal concretions. (I) Fluorescein staining of punctate epithelial erosions. (J) Punctal plug in inferior punctum. (K) Rubeosis iridis. (L) Blepharitis with marginal telangiectatic blood vessels. (M) Vernal keratoconjunctivitis with limbal disease. (N) Posterior polar cataract. (O) Hordeolum of the lower eyelid.

Of the 33 medical students included in this study, seven were females. A total of 165 slit-lamp recordings were successfully obtained (Figure 2), five by each medical student from 165 different patients on three different slit-lamp brands available in our service (Table 1). Captured ocular abnormalities included eyelid, ocular surface, corneal, anterior chamber, iris, and lenticular pathologies. Different smartphone device versions owned by medical students were used in this study which included iOS and Android smartphones (Table 2). All smartphone devices tested were compatible with this adapter for slit-lamp photography, and the application process was straightforward without noticeable technical difficulties.

**Table 1:** Slit lamp types tested in this study presented as numbers and percentages.

Slit lamp type	N (%)
Haag Streit BQ 900	6 (46.15%)
Huvitz Slit Lamp HS-5000	5 (38.46%)
Topcon SL-3G Slit Lamp	2 (15.38%)

**Table 2:** Smartphone types used in this study presented as numbers and percentages.

Smartphone types	N (%)
iPhone	
iPhone 13 Pro	2 (6%)
iPhone 13	4 (12%)
iPhone 12 Pro	1 (3%)
iPhone 12	8 (24%)
iPhone 11	3 (9%)
iPhone X	4 (12%)
Android	
Samsung	4 (12%)
Huawei	7 (21%)

The time used for the application of the adapter until the image capture ranged from 6 to 278 seconds (average = 39.51 ± 34.73 seconds). The time used for application improved gradually through the attempts from 67.3 ± 49.3 seconds for the first to 30.5 ± 25.7 seconds for the last recording task (Table 3). The learning process of the use of this slit-lamp photography technique using the adapter was perceived as relatively easy (2.2 ± 1). Medical students perceived the adapter used for slit-lamp photography to be potentially very valuable in clinical education (4.6 ± 0.75) and teleconsultations (4.7 ± 0.65).

**Table 3:** The time used by the medical students to complete each of the five recording tasks. Significance between periods was tested using the post hoc test (Dunn’s). *: Statistically significant at p ≤ 0.05. SD = standard deviation; Fr: Friedman test; p = p-value for comparing the task duration between the five recording attempts; p1 = p-value for comparing between first and each other task duration

Slit-lamp photography attempt	First	Second	Third	Fourth	Fifth	Fr	p
Mean ± SD	67.3 ± 49.3	39.7 ± 36.2	27.5 ± 14.2	32.7 ± 20.9	30.5 ± 25.7	40.748^*^	<0.001^*^
Median (minimum–maximum)	56 (20–278)	29 (7–206)	23 (9–69)	30 (7–122)	22 (6–108)		
p_1_	-	0.002^*^	<0.001^*^	<0.001^*^	<0.001^*^		

## Discussion

Ophthalmology referrals account for about 18.5-21% of outpatient referrals [12] and approximately 1% of emergency department visits [13], of which one-third are trauma-related [14]. Many general practitioners and emergency medicine specialists feel uncomfortable using ophthalmic instruments such as slit lamps and dilating drops because they think that their pre-graduate ophthalmic education was insufficient, even though such basic ophthalmic equipment is easily accessible [15-17].

Photographic telemedical consultations have been proven effective in several ophthalmic disorders such as assessment of glaucomatous optic neuropathy [7], grading of diabetic retinopathy [8], evaluation of senile cataracts [9], and postoperative cataract surgery imaging [4], provided the healthcare professionals have the basic skills of operating a slit lamp and can capture slit-lamp photographs. Hence, this study included medical students who had completed or were in ophthalmology rotation at the time of the study. All study participants were offered additional theoretical and practice sessions on slit lamp basic examination techniques before participation in this study.

To capture slit-lamp photos using a digital camera, the camera should be held stable with perfect alignment with the optical axis of one of the oculars of the slit lamp. Although there are several slit-lamp smartphone adapters, they are usually phone and microscope-specific which limits their use. The slit-lamp adapter used in this study consists of a metal body and a magnet (Figure 1). The metal body has a circular opening which can be manually widened by pulling the two blades apart enough to accommodate the diameter of the slit-lamp ocular against the resistance of two pushing springs. Once the ocular of the slit lamp is properly engaged, the adapter is released, and it will remain self-retained owing to the counteraction of the two springs (Video 1). The smartphone can then be snapped to the adapter using a preloaded ferromagnetic plate which can either be stuck on the phone using an adhesive or be stored between the phone body and its case. The smartphone camera can then be aligned with the optical axis of the slit lamp by following the light circle emanating from the slit-lamp ocular on the smartphone monitor (Video 1). If needed, the smartphone can be glided gently over the magnet to achieve the required centration of the view.

Medical students rated this slit-lamp photography technique as an easily acquired skill (2.2 ± 1). Although these results suggest that “primordial” future physicians can easily acquire the skill of smartphone slit-lamp photography, these results may vary depending on the slit-lamp adapter used. The smartphone adapter used in this study worked universally with all tested slit lamps (Table 1). The socket for the slit-lamp ocular needs to be widened manually against the pressure of the metal springs (Figure 1) to accommodate the diameter of the cylinder of the ocular of the slit lamp, following which it will be released to stay stable and self-retainable for efficient application of the smartphone. This adapter also worked universally with all tested smartphone brands and versions obtained by the included medical students (Table 2). Because of the magnetic attachment, when the metal plate is attached to the phone at a suitable distance from the camera, the phone will snap to the adapter, and with fine movements, the camera will be aligned with the slit-lamp ocular. The application was straightforward for all study participants who could master it in a time-efficient way over the five attempts at photography (Table 3).

Based on their current stage of medical education and training, this technique of smartphone slit-lamp photography was perceived by the study participants as of high potential value in clinical education (4.6 ± 0.75) and teleconsultations (4.7 ± 0.65). To our knowledge, this is the first study of medical students’ perspectives regarding smartphone slit-lamp photography.

Limitations of this study include the omission of photography of the posterior segment. Although slit-lamp examination and photography are a relatively easily acquirable skill for medical students based on our prior observations and this study, our anecdotal observations show that slit-lamp biomicroscopic examination of the posterior segment is a relatively difficult task for medical students, and slit-lamp photography in such examinations is even more difficult. Students preferred direct and panoptic ophthalmoscopes to the slit-lamp biomicroscopic retinal examination. We think that this can be investigated separately in a future study.

## Conclusions

Based on this study, educating medical students on slit-lamp photography is a relatively easy and acquirable skill. The use of this skill during their education can be a valuable asset to their medical education. Acquiring this skill can help them with their future careers in terms of telemedical, clinical, and ophthalmic consultation when needed. Future directions may include evaluation of the accuracy and utility of this method of slit-lamp photography in telemedical diagnosis and the management of urgent ocular conditions such as microbial keratitis and ocular trauma.
